# Ramadan fasting for patients with chronic respiratory diseases: a systematic review and consensus recommendations for healthcare professionals

**DOI:** 10.1183/23120541.01102-2024

**Published:** 2025-09-22

**Authors:** Fasihul Khan, Sameen Toor, Rayid Abdulqawi, Huzaifa Adamali, James D. Chalmers, Nazia Chaudhuri, Nazim Ghouri, R. Gisli Jenkins, Anna Murphy, Najib Rahman, Rafaqat Rashid, Imran Satia, Salman Siddiqui, Salman Waqar

**Affiliations:** 1 Dept of Respiratory Medicine, University Hospitals of Leicester NHS Trust, Leicester, UK; 2 King Faisal Specialist Hospital and Research Centre & College of Medicine, AlFaisal University, Riyadh, Saudi Arabia; 3 Bristol Interstitial Lung Disease Service, Bristol, UK; 4 Division of Molecular and Clinical Medicine, University of Dundee, Ninewells Hospital and Medical School, Dundee, UK; 5 Ulster University, School of Medicine, Derry–Londonderry, UK; 6 School of Medicine, University of Glasgow and Queen Elizabeth University Hospital, Glasgow, UK; 7 National Heart and Lung Institute, Imperial College London, London, UK; 8 University of Oxford and Oxford Centre for Respiratory Medicine, Oxford, UK; 9 Al-Balagh Academy, Bradford, UK; 10 Dept of Medicine, McMaster University, Hamilton, Canada; 11 Firestone Institute for Respiratory Health, St Joseph's Hospital, Hamilton, Canada

## Abstract

**Background:**

Ramadan, observed by nearly 2 billion Muslims worldwide, involves fasting from dawn to sunset, which can present challenges for individuals with chronic respiratory diseases due to altered medication regimens and oral intake restrictions. This study aimed to synthesise current evidence and develop consensus recommendations for managing asthma, COPD, interstitial lung disease (ILD) and bronchiectasis during Ramadan.

**Methods:**

A comprehensive search of electronic databases including MEDLINE, Embase and Google Scholar was conducted following a pre-specified protocol (PROSPERO identifier number CRD42024532759) to identify studies on Ramadan fasting outcomes in individuals with chronic respiratory diseases. The findings informed consensus recommendations stratified by the risk of adverse outcomes using International Diabetes Federation and the Diabetes and Ramadan risk assessment criteria. An international expert group of medical and religious experts refined these guidelines, achieving consensus approval.

**Results:**

11 studies met the inclusion criteria, primarily addressing asthma and COPD, with no relevant studies on ILD or bronchiectasis. The studies indicated that fasting did not significantly impact hospitalisation rates or lung function tests in individuals with stable asthma and COPD. However, small sample sizes and methodological limitations restricted generalisability. 19 recommendations were developed to support patients considering fasting, emphasising pre-Ramadan consultations, individualised risk assessments, and adjustments to medication regimens.

**Conclusion:**

This systematic review highlights the need for larger, well-designed studies to understand Ramadan fasting implications across chronic respiratory diseases. The developed recommendations provide a structured approach to assess fasting risks, ensuring informed and safe guidance during Ramadan. Future research should address identified gaps, supporting evidence-based guidelines that reconcile medical and religious considerations.

## Shareable abstract

**This article reviews the existing literature and offers culturally sensitive guidelines for managing chronic respiratory diseases during Ramadan fasting, highlighting expert consensus and practical recommendations for individualised patient care** https://bit.ly/4hESar3

## Introduction

Ramadan, the holiest month in the Islamic lunar calendar, is observed by almost 2 billion Muslims worldwide. During this month, Muslims fast from dawn (suhoor) to sunset (iftar), abstaining from food, drink and medications including oral, inhaled or intranasal forms [1]. Fasting duration varies by geographical location, with longer periods in higher latitudes. In the United Kingdom (UK) and northern Europe, fasting can exceed 20 h during summer, with much shorter durations in the winter months [2]. Outside fasting hours, Muslims resume normal consumption of food, drink and medications.

Religious exemptions are granted to high-risk groups, including the frail, elderly, children, pregnant or lactating women, and those with significant health conditions [3]. Those with acute or chronic illnesses exacerbated by fasting are also exempt. Alternatives for those unable to fast include nonconsecutive fasting, making up missed fasts during winter, or offering a charitable donation (fidyah). However, some high-risk individuals may still choose to fast for personal reasons, such as spiritual growth, solidarity with the fasting community, habitual practice or as a means of overcoming personal challenges [4]. Consulting healthcare professionals and trusted Islamic scholars is crucial for these individuals to understand potential risks and align with medical and spiritual guidance.

Respiratory diseases affect >500 million people globally, presenting significant challenges during Ramadan [5]. The effects of fasting and altered medication patterns on chronic lung diseases remains poorly understood, with an absence of robust studies and formal guidelines. Urgent action is needed to develop expert consensus statements and culturally sensitive research to inform clinicians’ practice. Collaboration with religious leaders is essential to provide clarity and ensure a patient-centred approach that respects spiritual aspirations while safeguarding respiratory health.

This article addresses this gap through a systematic review of existing literature evaluating the effects of fasting in patients with chronic respiratory disease, leading to consensus-based recommendations. This effort, building on a review led by the British Islamic Medical Association during the coronavirus disease 2019 pandemic, involves healthcare professionals and religious scholars (https://britishima.org/ramadan/). The aim is to support clinicians with the knowledge and tools to counsel and support patients with chronic respiratory disease during Ramadan, focusing on COPD, asthma, interstitial lung disease (ILD) and bronchiectasis [5].

## Methods

This systematic review followed a pre-specified protocol (PROSPERO registration number CRD42024532759) and the Preferred Reporting Items for Systematic Reviews and Meta-Analyses guidelines [6]. A search of MEDLINE (1946 to date), Embase (1974 to date) and Google Scholar, last conducted on 9 April 2024, included broad search terms for chronic respiratory diseases (COPD, asthma, ILD, bronchiectasis) and Ramadan fasting (the MEDLINE search strategy is presented in the supplementary material). Unpublished studies were identified *via* medRxiv and bioRxiv. Two reviewers (F. Khan, S. Toor) independently screened titles, abstracts and full texts. Discrepancies were resolved by consensus. Reference lists were also searched. Conference abstracts that provided sufficient detail were considered. Inclusion criteria encompassed original studies documenting clinical outcomes in fasting patients aged >18 years with chronic respiratory diseases. There were no restrictions on language, study type, sample size or the publication year. Exclusion criteria applied to studies not addressing fasting outcomes in diagnosed respiratory conditions.

Data extraction followed a structured proforma, validated by a second reviewer and included study design, sample size, geographic location, diagnostic criteria, participant demographics and clinical outcomes (hospitalisation rates, exacerbation, lung function, health-related quality of life (HRQoL) scores, biomarkers and medication adherence). The risk of bias was assessed in duplicate using the Newcastle–Ottawa Scale (NOS) [7], which evaluates key areas such as the selection of study groups, their comparability and the ascertainment of outcomes. A modified version was utilised to assess the risk of bias for cross-sectional studies [8]. All studies were including regardless of bias rating.

Eligible studies were analysed narratively to identify trends, inconsistencies and gaps. Summary tables presented key study and participant characteristics. Findings were combined with clinical expertise to develop consensus statements, categorised by adverse outcomes risk using International Diabetes Federation and the Diabetes and Ramadan criteria [9]. A three-tier risk assessment approach was adopted, recommending fasting support for low-to-moderate risk after treatment optimisation, while advising high-risk individuals to consider alternatives such as fidyah or making up their fasts after Ramadan once their disease control is more stable. We also compiled a summary of long-acting inhaled therapies to support adherence during fasting hours.

Consensus statements were drafted by a core group of respiratory experts, experienced in managing Muslim patients observing Ramadan. These initial statements were then disseminated to a wider panel of experts for approval and feedback. Each panel member reviewed the statements and participated in a structured discussion. The decision-making process was structured around a formal voting system, in which all reviewers voted on each statement. Based on the feedback gathered, the consensus statements were revised to incorporate the collective advice of the experts. In cases where consensus was not reached in the first round, the statements were revised based on panel feedback, and a second round of voting occurred. This iterative process ensured that the final recommendations were robust and representative of collective expert opinion.

## Results and discussion

**FIGURE 1:**
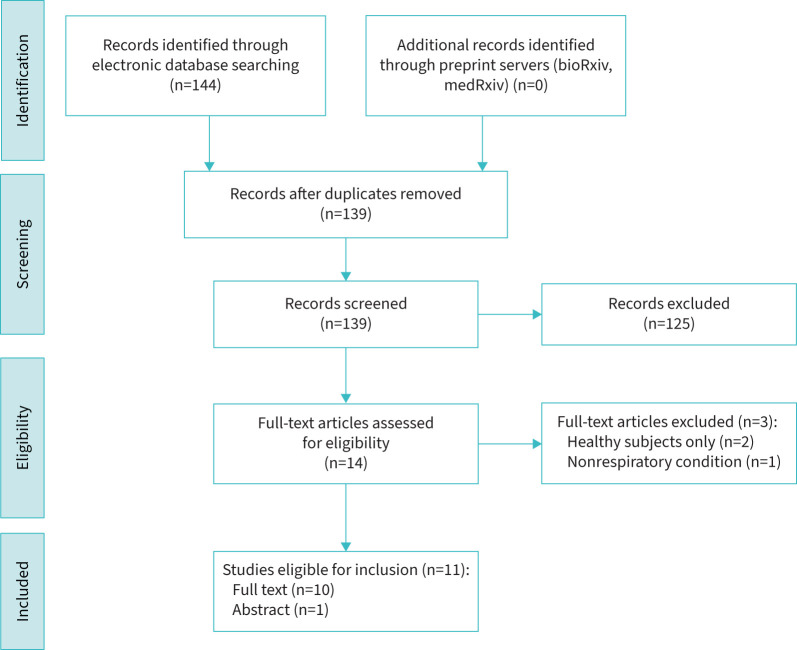
Preferred Reporting Items for Systematic Reviews and Meta-Analyses flow diagram illustrating systematic search and screening strategy, including numbers of studies meeting eligibility criteria and numbers excluded.

Searches on 9 April 2024 yielded 144 studies. After removing duplicates and screening, 10 full-text articles and one abstract, published worldwide between 2004 and 2022, were included covering COPD (n=5) and asthma (n=7) (table 1). No studies on ILD or bronchiectasis were identified (figure 1). A substantial number of articles discussing health outcomes during Ramadan for individuals without specific health conditions were excluded. No randomised controlled trials were found, and most studies originated from the Middle East. The NOS risk-of-bias assessment highlighted potential biases related to the selection and comparability of cohorts (table 2). All studies were from single centres, and therefore the external validity of relevant findings is lacking. Comparability between fasting and nonfasting groups was frequently unclear, as no studies controlled for baseline differences such as disease severity or comorbidities. Outcome measures were robust for clinical end-points, but relied on self-reporting for medication adherence, introducing subjectivity. Small sample sizes and regional focus raised concerns about the generalisability of the results. Included studies frequently lacked information on fasting duration, which can vary significantly based on geography and season, ranging from ∼10–12 h in lower latitudes to 18–20 h during summer months. This variability can significantly impact the interpretation and applicability of findings.

**TABLE 1:** Summary of included studies

First author, year [reference]	Country	Centre	Study design	Condition	Diagnostic criteria	Sample size	Controls	Male %	Outcomes reported	Fasting duration
**Adeli, 2015 [** 10 **]**	Iran	Single	Prospective	Asthma	Moderate–severe (not otherwise specified)	30	NA	43	ACQ and spirometry; pre-, during and post-Ramadan	Not specified
**Askari, 2016 [** 11 **]**	Iran	Single	Prospective	Asthma	Moderate–severe (GINA 2008)	15	14	ND	Blood biomarkers, symptoms, and spirometry; before and after Ramadan	Not specified
**Aydin, 2014 [** 12 **]**	Turkey	Single	Cross-sectional	Asthma COPD	GINA 2009 GOLD 2008	150 150	NA NA	13 79	Inhaler adherence	Not specified
**Bener, 2006 [** 13 **]^#^**	Qatar	Single	Prospective	Asthma	Not specified	754	NA	ND	Hospitalisation rates and spirometry; pre-, during and post-Ramadan	Not specified
**Erkekol, 2006 [** 14 **]**	Turkey	Single	Cross-sectional	Asthma	GINA 2003	121	NA	17	Attendance at follow-up visits, diagnostic tests and medication use	Not specified
**Ghaffary, 2022 [** 15 **]**	Iran	Single	Prospective	Asthma	Moderate–severe (GINA)	60	60	67	Symptoms and spirometry; pre- and post-Ramadan	Not specified
**Mekki, 2022 [** 16 **]**	Tunisia	Single	Prospective	COPD	Stable (GOLD 2019)	20	NA	100	VQ11, 6MWT and spirometry; weeks 1, 2 and 4 of Ramadan	∼16.5 h
**Mrad, 2019 [** 17 **]**	Tunisia	Single	Prospective	COPD	Stable (GOLD 2019)	15	NA	100	Blood biomarkers; pre-, during and post-Ramadan	∼16.5 h
**Norouzy, 2013 [** 18 **]**	Iran	Single	Prospective	Asthma	Stable (GINA year not specified)	29	NA	34	Symptoms, PEFR and spirometry; pre- and post-Ramadan	∼14 h
**Rejeb, 2018 [** 19 **]**	Tunisia	Single	Prospective	COPD	GOLD 2017	15	NA	100	Blood biomarkers and spirometry; pre-, during and post-Ramadan	∼16.5 h
**Zouari, 2018 [** 20 **]**	Tunisia	Single	Prospective	COPD	GOLD 2017	16	NA	100	Spirometry; pre-, during and post-Ramadan	∼16.5 h

Data are presented as n, unless otherwise stated. NA: not applicable; ACQ: Asthma Control Questionnaire; GINA: Global Initiative for Asthma; ND: not defined; GOLD: Global Initiative for Chronic Obstructive Lung Disease; 6MWT: 6-min walk test; PEFR: peak expiratory flow rate. **^#^**: abstract only.

**TABLE 2:** Risk-of-bias assessment was rated using the Newcastle–Ottawa Scale, with studies rated as poor, fair or good

First author, year [reference]	Study design	Selection	Comparability	Outcome	Total score	Quality
**Adeli, 2015 [** 10 **]**	Prospective cohort	⋆⋆⋆		⋆⋆	5	Poor
**Askari, 2016 [** 11 **]**	Prospective cohort	⋆⋆⋆⋆	⋆	⋆⋆	7	Good
**Aydin, 2014 [** 12 **]**	Cross-sectional	⋆⋆⋆		⋆⋆	5	Poor
**Bener, 2006 [** 13 **]^#^**	Prospective cohort	⋆⋆⋆⋆	⋆	⋆⋆	7	Good
**Erkekol, 2006 [** 14 **]**	Cross-sectional	⋆⋆⋆		⋆	4	Poor
**Ghaffary, 2022 [** 15 **]**	Prospective cohort	⋆⋆⋆⋆	⋆	⋆⋆	7	Good
**Mekki, 2022 [** 16 **]**	Prospective cohort	⋆⋆⋆		⋆⋆	5	Poor
**Mrad, 2019 [** 17 **]**	Prospective cohort	⋆⋆⋆		⋆⋆	5	Poor
**Norouzy, 2013 [** 18 **]**	Prospective cohort	⋆⋆⋆		⋆⋆⋆	6	Poor
**Rejeb, 2018 [** 19 **]**	Prospective cohort	⋆⋆⋆		⋆⋆⋆	6	Poor
**Zouari, 2018 [** 20 **]**	Prospective cohort	⋆⋆⋆		⋆⋆⋆	6	Poor

**^#^**: abstract only.

A panel of 12 expert reviewers from UK, North America and the Middle East developed consensus recommendations, ensuring diverse representation. The panel included both Muslim and non-Muslim experts, respiratory physicians, general practitioners, religious scholars and a pharmacist, ensuring that the recommendations were both religiously sensitive and medically robust.

### Asthma

Seven studies with 1159 participants with asthma who observed fasting were included [10–15, 18]. Despite methodological differences, findings on spirometry, HRQoL scores and hospitalisation rates were consistent. A prospective cohort study in Qatar (754 stable participants) found no increased hospitalisations or changes in lung parameters during Ramadan [13]. Similarly, smaller studies showed no significant changes in lung function or Asthma Control Questionnaire scores before and after Ramadan [10, 11, 15, 18]. However, details on how patients adapted their therapies during Ramadan were lacking.

Two behavioural studies as described by the authors, conducted in Turkey, evaluated medication adjustments during Ramadan fasting. One study involving 150 adults found that 65.3% opted to fast during Ramadan, with 81% reporting no difficulty adjusting therapies to suhoor and iftar timings [12]. Another study showed that 87% of fasting asthmatics successfully rearranged their medication schedules, enabling them to fast without major issues [14].

### COPD

Four studies from a single Tunisian institute with a total of 66 participants with stable COPD were identified [16, 17, 19, 20], supplemented by an additional behavioural study, as reported by the authors, involving 150 stable COPD patients [12]. A study of 20 nonsmoking stable COPD patients found significant spirometric worsening during the second week of Ramadan (forced expiratory volume in 1 s (FEV_1_) 48±22% predicted; forced vital capacity (FVC) 66±13% pred) compared with measurements prior to Ramadan (FEV_1_ 56±21% pred; FVC 79±12% pred), but appeared to recover by week four (FEV_1_ 54±22% pred; FVC 73±11% pred) [16]. These findings were not consistent with two earlier studies of 15 and 16 stable COPD male patients, where no changes were observed in spirometric values between baseline and week 2 [18, 20]. Inflammatory biomarkers and oxidative/antioxidative stress biomarkers showed no substantial change [17].

In a study evaluating patient behaviours, 92.6% of patients with COPD chose to fast during Ramadan, with a significant majority (90.6%) completely discontinuing their regular inhaled therapy [12]. Notably, studies reporting hospitalisation or exacerbation data were not identified. In the sole study assessing quality-of-life scores, mean VQ11 total scores increased from 22.5±4.0 at baseline, to 38.5±3.2 at week 4, indicating heightened symptom burden during Ramadan fasting [16].

### ILD and bronchiectasis

No published studies on the effects of fasting in patients with ILD or bronchiectasis were identified.

### Summary of evidence

The evidence suggests no significant adverse health outcomes from Ramadan fasting in stable patients with asthma and COPD, but data are limited and methodologically constrained. Medication adjustments for fasting patients with asthma appears to be effective, while COPD patients tend to forgo regular treatments, raising management concerns. The lack of studies on ILD and bronchiectasis underscores the critical need for comprehensive research to guide clinical practice effectively.

#### General recommendations

**FIGURE 2:**
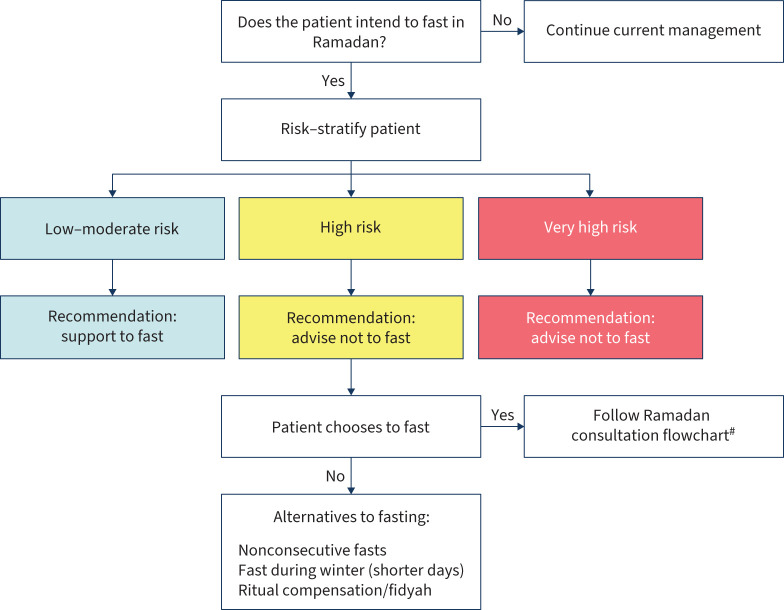
Proposed risk stratification and decision-making flowchart for clinicians assessing the suitability of patients with chronic respiratory diseases for fasting during Ramadan. ^#^: figure 3.

Published studies provide limited evidence to inform recommendations for patients with chronic respiratory diseases who wish to fast during Ramadan. Therefore, a comprehensive, individualised pre-Ramadan consultation is crucial for all such patients (figure 2).

**FIGURE 3:**
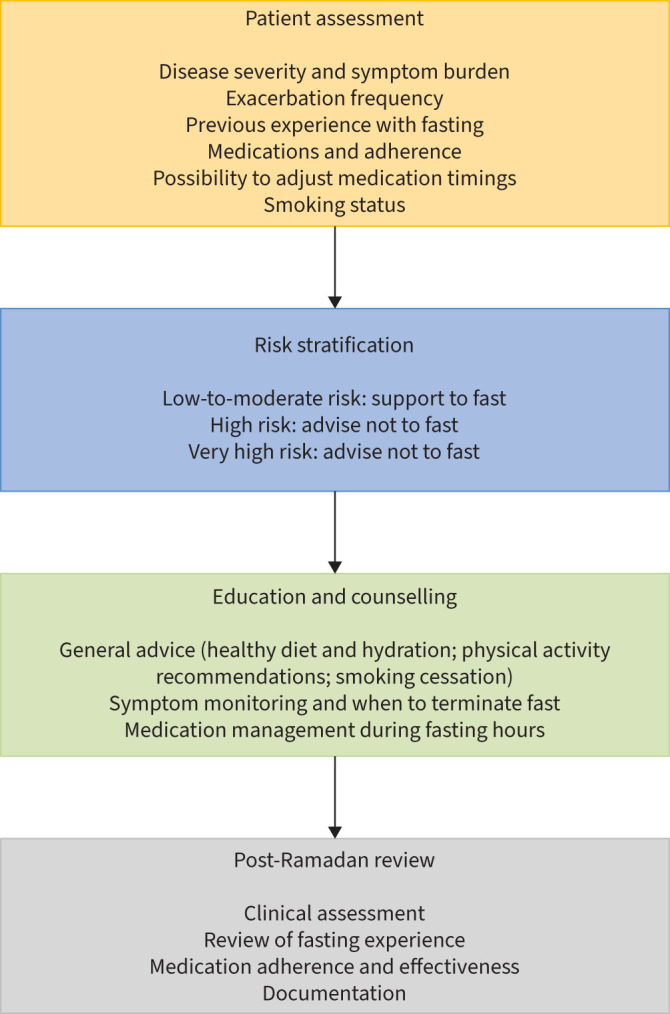
Flowchart for pre-Ramadan consultation, illustrating a structured approach for healthcare professionals to assess and manage patients with chronic respiratory diseases considering fasting during Ramadan.

During this consultation, individual risk should be assessed (figure 3) considering previous fasting experiences, symptom burden, comorbidities and disease severity markers such as pulmonary function tests. Tailored education should be provided, covering health eating habits, adequate fluid intake, weight management and gentle physical activities such as walking or stretching, preferably scheduled during nonfasting hours. Several studies have reported successful outcomes from Ramadan smoking cessation initiatives [21–23], and given that smoking violates the fast, Ramadan offers a unique opportunity to promote cessation strategies, including referral to stop-smoking services and the use of nicotine patches.

Comorbidities such as cardiovascular disease, diabetes and obesity can significantly impact the patient's overall risk profile during Ramadan fasting. These comorbid conditions, often present in patients with chronic respiratory disease, should be factored into the pre-Ramadan risk assessment. For example, patients with heart failure or diabetes may face additional complications related to hydration status or glucose control, which may exacerbate respiratory symptoms.

Emphasis should be placed on medication adherence, particularly for inhaler and oral medication regimens. Patients should be advised on the importance of terminating their fasts if they experience any health deterioration, consider inhaler/nebulised therapy as appropriate, and seek medical attention as necessary. This holistic approach safeguards patient health and enhances understanding and management of respiratory conditions during Ramadan.

Another crucial consideration is the allowance for trial fasts. Ideally, individuals should conduct these trials before Ramadan to gauge their capacity for fasting and terminate if discomfort occurs without repercussions. Trial fasts can also be conducted during Ramadan, particularly for high-risk patients determined to fast, to evaluate their suitability for fasting.

#### Recommendations in asthma

Evidence suggests that Ramadan fasting among patients with stable asthma does not lead to increased hospitalisation, greater disease severity or physiological deterioration. Asthma patients seem receptive to modifying inhaler therapy during Ramadan, providing an opportunity for healthcare professionals to discuss the optimal balance between medication adherence and maintaining disease control. Importantly, for patients on maintenance and reliver therapy (MART), transitioning to a fixed regimen should be considered in order to simplify dosing schedules and enhance compliance.

- Prior to determining suitability for fasting in Ramadan, all asthma patients should be provided with up-to-date personalised action plans, including an assessment of treatment adherence and optimisation of inhaler technique.
- Adjustments to maintenance inhaler regimens to fasting-compatible once or twice daily doses (table 3) should ideally be made ≥4 weeks prior to Ramadan, with prompt patient-initiated review in the event of deterioration. For patients on MART regimens, transitioning to a fixed regimen should be considered.
- Stable asthmatics characterised by well-controlled symptoms, one or fewer exacerbations in the past year, no-life threatening exacerbations in the past year, and no exacerbations within 3 months before the start of Ramadan may be considered low-risk and should be supported to fast.
- Individuals with poorly controlled symptoms requiring frequent use of short-acting bronchodilators or additional MART (inhaled corticosteroid (ICS)/fast-acting long-acting β_2_-agonist as reliever) therapy during the day, or those dependent on systemic corticosteroids are classified as high-risk and may be advised not to fast.
- Individuals on biologic therapies who are stable, well-controlled without an exacerbation in the past 3 months should be supported to fast. The acceptability of subcutaneous therapies during fasting hours varies among Muslim scholars. Therefore, the decision to fast should be left to the individual patient, allowing them to follow their personal religious beliefs.

**TABLE 3:** Overview of once-daily inhalers available for the management of asthma and COPD

Type of inhaler device	Common brand names
**LABA**	
Aerosol	Striverdi Respimat
Dry powder	Onbrez Breezhaler
**LAMA**	
Aerosol	Spiriva Respimat
Dry powder	Seebri Breezhaler, Braltus Zonda, Spiriva HandiHaler, Incruse Ellipta
**Combination LABA/LAMA**	
Aerosol	Spiolto Respimat
Dry powder	Ultibro Breezhaler, Anoro Ellipta
**ICS**	
Aerosol	Alvesco MDI
Dry powder	Asmanex Twisthaler
**Combination ICS/LABA**	
Aerosol	NA
Dry powder	Relvar Ellipta
**Combination ICS/LABA/LAMA**	
Aerosol	NA
Dry powder	Enerzair Breezhaler, Trelegy Ellipta

LABA: long-acting β_2_-agonist; LAMA: long-acting muscarinic antagonist; ICS: inhaled corticosteroid; MDI: metered dose inhaler; NA: not applicable.

#### Recommendations in COPD

Current evidence does not definitively determine whether fasting exacerbates hospitalisation rates, disease severity or symptom burden in COPD patients. However, adherence to inhaled therapy appears to be lower compared with asthmatics, although this finding requires further validation.

- Prior to determining suitability for fasting during Ramadan, all COPD patients should be provided with up-to-date personalised management plans [23], which include assessing treatment adherence and inhaler technique optimisation.
- If changes are made to maintenance inhaler regimens to support fasting (table 3), these should be made ≥4 weeks prior to Ramadan.
- Individuals with COPD Global Initiative for Chronic Obstructive Lung Disease (GOLD) 2023 group A [24] characterised by low symptom burden (modified Medical Research Council (mMRC) dyspnoea score 0 or 1), low risk of exacerbations (0 or 1 exacerbations in the past year that did not lead to hospital admission) are deemed low-risk and may be supported to fast.
- Individuals with COPD GOLD 2023 group B, characterised by increased symptom burden (mMRC dyspnoea score ≥2) with greater reliance on short-acting bronchodilators, are considered high-risk and may be advised not to fast. If such patients wish to fast, long-acting bronchodilator treatment should be optimised to minimise requirement for short-acting bronchodilators during fasting hours.
- Individuals with COPD GOLD 2023 group E characterised by frequent exacerbations (two or more outpatient visits or one or more hospitalisations), are categorised as very high risk and may be advised not to fast.

#### Inhalers in small airways disease

The use of inhalers or nebulisers during Ramadan remains debated among Islamic scholars. The prevailing opinion is that inhaler therapy invalidates the fast because a small proportion of aerosolised particles or vapours from inhalers enter the throat and subsequently the stomach. To improve adherence and minimise disruptions to treatment, it is recommended that inhaler usage be scheduled between iftar (sunset) and suhoor (dawn). Once-daily scheduling is particularly necessary during the summer months or in regions where the fasting day is lengthy. Table 3 outlines recommended inhalers that are suitable for such regimens during fasting periods. This list is not exhaustive, and prescribers should ensure that the selected inhalers are approved by their local formulary guidelines.

#### Recommendations in ILD

Since ILD represents a heterogenous group of conditions, these expert recommendations should be adapted based on the specific subtype and severity of disease.

- Individuals with mild or stable ILD, characterised by well-controlled symptoms and no recent exacerbations, are considered low risk and should be supported to fast.
- Individuals receiving antifibrotic and/or immunomodulatory therapies that cannot be adequately adjusted to fit the fasting schedule are considered high risk and may be advised not to fast.
- Individuals with severe disease, who require increased frequency of nutritional intake, are considered high risk and may be advised not to fast.
- Individuals who are on medications for symptom control including opiates and anxiolytics are considered high risk and may be advised not to fast.
- Individuals with severe disease characterised by high symptom burden, FVC <50% pred, a progressive fibrotic phenotype, long-term or ambulatory oxygen therapy or history of recent exacerbation (<3 months) are categorised as very high risk and may be advised not to fast.

#### Recommendations in bronchiectasis

Patients with bronchiectasis who experience chronic airway colonisation, particularly with organisms such as *Pseudomonas aeruginosa*, should be assessed carefully before considering fasting. While these patients may not experience exacerbations once stabilised, the presence of chronic infections increases the complexity of fasting-related decisions.

- All patients should be advised to modify their airway clearance regimes to ensure continuation during Ramadan, while maintaining adequate hydration and physical activity to support airway clearance.
- Individuals with stable bronchiectasis, who have infrequent exacerbations and can adjust their medication timing, are low risk and should be supported to fast.
- Individuals with frequent exacerbations, recent hospitalisation (<3 months), or are unable to adjust the timing of inhaled or nebulised long-term antibiotics, are classified as high risk and may be advised not to fast.
- Individuals experiencing an exacerbation requiring oral or intravenous antibiotics are very high risk and may be advised not to fast.

### Religious considerations

During Ramadan, certain medical therapies do not invalidate the fast and can be continued as usual. These include supplemental home oxygen therapy, continuous positive airway pressure therapy and noninvasive ventilation, vaccinations or patches (nicotine and others). There remains a debate among Islamic scholars regarding the permissibility of non-nutritive injections (subcutaneous, intramuscular and intravenous) while fasting, and thus patients should be advised to consult with their trusted scholars.

Additionally, the use of airway clearance devices, such as positive expiratory pressure, is permissible during fasting, since they only deliver pressurised air to the lungs. This important clarification enables patients to maintain their airway clearance routines during Ramadan.

## Conclusion

This systematic review highlights a significant limitation in the current evidence base, as the included studies are single-centre, observational, and many are rated as poor on the NOS. We acknowledge that the recommendations presented in the manuscript are primarily based on expert consensus and not directly derived from the systematic review findings.

Consensus-based recommendations are designed to support healthcare providers by providing structured guidance on medication timing, lifestyle adjustments and risk assessments to optimise patient outcomes. They serve as informative recommendations and are not intended to be an authority or directive for healthcare professionals. The application of these recommendations should consider individual clinical judgement, specific circumstances and patient preferences. Future research should address gaps to support evidence-based guidelines, reconciling medical and religious considerations, enabling well-informed patient decisions. This collaborative effort between healthcare providers and religious scholars is essential to develop inclusive, practical and culturally sensitive fasting guidelines that safeguard patient health and spiritual wellbeing.

## Supplementary material

**Please note:** supplementary material is not edited by the Editorial Office, and is uploaded as it has been supplied by the author. Supplementary material 01102-2024.SUPPLEMENT
