# Supplementary material for: Ramadan fasting for patients with chronic respiratory diseases: a systematic review and consensus recommendations for healthcare professionals
Source: ERJ Open Res. 2025 Sep 22;11(5):01102-2024. doi: 10.1183/23120541.01102-2024 (PMC12451581; doi:10.1183/23120541.01102-2024)
Supplement: Supplementary file 1 [file 01102-2024.SUPPLEMENT.pdf]

Supplementary material

Supplementary Figure S1- Ovid MEDLINE(R) search strategy (last search carried out 9th April 2024)

Participants	Participants	Participants	Participants	Intervention
1. interstitial lung disease.mp. or Lung Diseases, Interstitial/	9. Langerhans cell histiocytosis.mp. or Histiocytosis, Langerhans-Cell/	20. berylliosis.mp. or Berylliosis/	31. Pulmonary embolism.mp. or Pulmonary Embolism/	40. ramadhan.mp. or Islam/
2. idiopathic pulmonary fibrosis.mp. or Pulmonary Fibrosis/ or Idiopathic Pulmonary Fibrosis/	10. Birt-Hogg-Dube Syndrome/ or Cystic lung disease.mp. or Lymphangioleiomyomatosis/	21. silicosis.mp. or Silicosis/	32. Cor pulmonale.mp. or Pulmonary Heart Disease/	41. ramadan.mp.
3. fibrosing alveolitis.mp.	11. Asbestos*.mp.	22. Chronic bronchitis.mp. or Bronchitis, Chronic/	33. Pleural effusion.mp. or Pleural Effusion/	42. ramad*.mp.
4. usual interstitial pneumonia.mp.	12. Asbestosis/	23. Chronic obstructive pulmonary disease.mp. or Pulmonary Disease, Chronic Obstructive/	34. Pneumothorax.mp. or Pneumothorax/	43. ramdan.mp.
5. progressive pulmonary fibrosis.mp.	13. Hypersensitivity pneumonitis.mp. or Alveolitis, Extrinsic Allergic/	24. Pulmonary Emphysema/ or Pulmonary Disease, Chronic Obstructive/ or Lung Diseases, Obstructive/ or Chronic obstructive airways disease.mp.	35. Nintedanib.mp.	44. ramdhan.mp.
6. progressive fibrosing interstitial lung disease.mp.	14. Extrinsic allergic alveolitis.mp.	25. asthma.mp. or Asthma, Occupational/ or Asthma, Exercise-Induced/ or Asthma/ or Cough-Variant Asthma/ or Asthma-Chronic Obstructive Pulmonary Disease Overlap Syndrome/	36. Pirfenidone.mp.	45. ramazan.mp.
7. non-specific interstitial pneumonia.mp.	15. pneumonitis.mp. or Pneumonia/	26. bronchi*.mp.	37. Cocaine Smoking/ or Water Pipe Smoking/ or Smoking/ or Marijuana Smoking/ or Cigarette Smoking/ or Smoking.mp. or Pipe Smoking/ or Tobacco Smoking/ or Smoking Cessation/ or Smoking Water Pipes/ or Smoking Cessation Agents/ or Smoking, Non-Tobacco Products/or Smoking Devices/ or Smoking Prevention/ or Cigar Smoking	46. iftar.mp.
8. interstitial pneumonia.mp.	16. Sarcoidosis/ or sarcoid.mp.	27. bronchiectasis.mp. or Bronchiectasis/	38. tobacco.mp	47. suhoor.mp.
	17. Granulomatous*.mp.	28. Sleep Apnea, Obstructive/ or Obstructive sleep apn*.mp.	39. 1 or 2 or 3 or 4 or 5 or 6 or 7 or 8 or 9 or 10 or 11 or 12 or 13 or 14 or 15 or 16 or 17 or 18 or 19 or 20 or 21 or 22 or 23 or 24 or 25 or 26 or 27 or 28 or 29 or 30 or 31 or 32 or 33 or 34 or 35 or 36 or 37 or 38	48. iftaari.mp.
	18. Granulomatous Disease, Chronic/	29. Obesity Hypoventilation Syndrome/		49. sehri.mp.
	19. Tuberculosis/ or tuberculosis.mp. or Mycobacterium tuberculosis/ or Tuberculosis, Pulmonary/	30. Pulmonary hypertension.mp. or Hypertension, Pulmonary/		50. 40 or 41 or 42 or 43 or 44 or 45 or 46 or 47 or 48 or 49
				51. 39 and 50
